# Fusing the 3’UTR of seed storage protein genes leads to massive recombinant protein accumulation in seeds

**DOI:** 10.1038/s41598-023-39356-3

**Published:** 2023-07-27

**Authors:** Masatake Kanai, Masaya Sugiyama, Maki Kondo, Kenji Yamada, Mikio Nishimura, Shoji Mano

**Affiliations:** 1 Laboratory of Organelle Regulation, National Institute for Basic Biology, Okazaki, 444-8585 Japan; 2 Department of Viral Pathogenesis and Controls, National Center for Global Health and Medicine, Ichikawa, 272-8516 Japan; 3 Department of Cell Biology, National Institute for Basic Biology, Okazaki, 444-8585 Japan; 4 Malopolska Centre of Biotechnology, Jagiellonian University, 30-387 Krakow, Poland; 5 Basic Biology Program, Graduate Institute for Advanced Studies, The Graduate University for Advanced Studies, SOKENDAI, Okazaki, 444-8585 Japan; 6 Present Address: Faculty of Science and Engineering, Konan University, Kobe, 658-8501 Japan

**Keywords:** Plant biotechnology, Plant cell biology

## Abstract

The demand for recombinant proteins is rising dramatically, and effective production systems are currently being developed. The production of recombinant proteins in plants is a promising approach due to its low cost and low risk of contamination of the proteins with endotoxins or infectious agents from the culture serum. Plant seeds primarily accumulate seed storage proteins (SSPs), which are transcribed and translated from a few genes; therefore, the mechanism underlying SSP accumulation has been studied to help devise ways to increase recombinant protein production. We found that the 3’UTR of SSP genes are essential for SSP accumulation and can be used in the production of recombinant proteins in *Arabidopsis*. Fusion of the 3’UTR of SSP genes to the 3’ ends of DNA sequences encoding recombinant proteins enables massive accumulation of recombinant proteins with enzymatic activity in *Arabidopsis* seeds. This method is also applicable to the production of human Interferon Lambda-3 (IFN-lambda 3), a candidate biopharmaceutical compound against hepatitis C infection. Considering the low cost and ease of protein production in *Arabidopsis*, as well as the rapid growth of this plant, our method is useful for large-scale preparation of recombinant proteins for both academic research and biopharmaceutical production.

## Introduction

Plant seeds accumulate large amounts of storage products to supply nutrients for germination and post-germinative growth. Seeds accumulate large amounts of starch and oils as energy sources for proteins as a source of amino acids. In plants, the protein content is much higher in seeds than in roots, leaves, stems, or other organs. Although high levels of proteins are present in seeds, there are only a limited number of protein species, which are referred to as seed storage proteins (SSPs). SSPs comprise the major proportion of proteins in seeds^1^, thus determining various properties, such as the nutritional value and processing characteristics of seeds^2,3^. Therefore, breeders have focused on SSP contents in crop seeds. Studies on SSPs have also helped to elucidate the intracellular mechanisms of protein transport and accumulation. Genetic studies in *Arabidopsis*, maize, rice, and so on have revealed many components of the SSP biosynthetic pathway^4,5^.

SSP biosynthesis has drawn increasing attention since the demand for biopharmaceutical proteins is rising dramatically^6–8^. Seed-based heterogenous protein production systems have been improved using information about the mechanisms underlying SSP transport and accumulation^9–13^. In fact, dozens of reports describe the production of biopharmaceutical proteins in seeds of various plant species. Interleukin-7, a cytokine involved in the regulation of lymphoid homeostasis, was produced in rice seeds^14^. Interleukin-10, a candidate biopharmaceutical for treating inflammatory allergy and autoimmune diseases, was produced in rice and *Arabidopsis* seeds^12,15,16^. These studies demonstrate that seed-based biopharmaceutical production systems are promising due to their low cost and high quality. However, these studies also raise the issue of low yields of biopharmaceutical proteins. SSPs are the predominant proteins produced in seeds, whereas the yields of biopharmaceutical proteins in seed-based systems are, in most cases, considerably smaller than those of SSPs. However, the key factors underlying the predominant accumulation of SSPs remain unclear.

In rice seeds, the 3' untranslated region (3’ UTR) has been reported to play a critical role in post-transcriptional regulation^17^. The SSPs in rice seeds, prolamine and glutelin, are known to be localized to the protein body ER and cisternal ER, respectively, and mRNA is correctly transported to the protein body ER or the cisternal ER. It has been reported that the localization of mRNAs encoding rice prolamine and glutelin is regulated by the 3'UTR^18,19^. In addition, studies on the synthesis and accumulation of useful recombinant proteins in rice seeds are promising, and the localization of recombinant proteins to the protein body ER has been shown to be an important strategy^12,13,20^. On the other hand, it has been reported that the 3'UTR itself is not effective in enhancing the accumulation of recombinant proteins in rice seeds^21^, and the function of the 3'UTR remains unclear.

In this study, we report that the 3’UTRs of SSP genes are a key factor in determining the predominant accumulation of SSPs. We also show that protein yield can be dramatically increased by fusing the 3’UTR of SSPs genes to any genes of interest. This method enables the production of biopharmaceuticals efficiently by changing them as a major proportion of proteins in the seeds.

## Results

### Transcripts of SSP genes are abundant but not dominant in seeds

**Figure 1:**
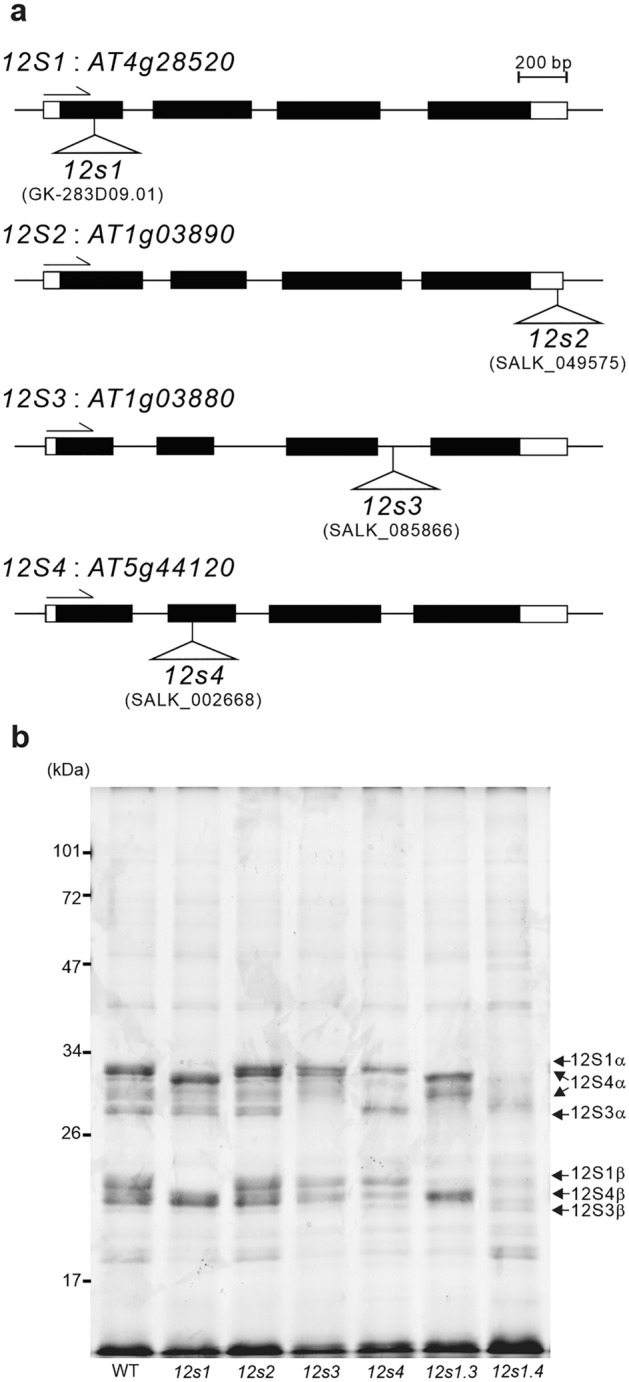
Establishment of single and double knockout lines of SSPs. (**a**) Schematic representation of the T-DNA insertion positions in *12s1*, *12s2*, *12s3*, and *12s4* genes. White and black boxes indicate the untranslated regions and the coding regions, respectively. Triangles show the position of T-DNA insertion in the knockout lines. Arrows show the direction of transcription. (**b**) SDS-PAGE analysis of seed proteins in the single and double knockout lines. Total protein was extracted using SDS sample buffer containing 10% (v/v) 2-mercaptoethanol. Total protein (10 µg) was loaded onto each well of a 12.5% polyacrylamide gel and protein bands were visualized by CBB staining. Arrows indicate the subunits of 12S globulins, which were separated using a reducing agent in the buffer. *12s1.3* and *12s1.4* are the knockout mutants of both *12S1* and *12S3*, and *12S1* and *12S4*, respectively.

Seeds predominantly accumulate a limited number of SSPs, and 80% of the total protein in *Arabidopsis thaliana* seeds consists of 12S globulin encoded by the *12S1*, *12S3*, and *12S4* genes and 2S albumin encoded by the *2S3* gene^22,23^. However, the mechanisms that regulate the predominant accumulation of SSPs remain unclear. To determine what drives the accumulation of SSPs, we focused on 12S globulins, the most abundant protein in *Arabidopsis* seeds, comprising approximately 70% of total proteins^22^. The single knockout mutants of *12S1*, *12S2*, *12S3* or *12S4* gene were established (Fig. 1A) and double knockout mutants of both *12S1* and *12S3* or both *12S1* and *12S4* were produced by crossing. It confirmed that 12S1, 12S3, and 12S4 are the major proteins that accumulate in the wild-type plant, and that their accumulation was dramatically reduced in each knockout mutant (Fig. 1B). It is reported that the accumulation of 12S2 in seeds was minor^22^, and the SDS-PAGE analysis of seed protein in established 12S2 T-DNA mutants was similar to that of the wild-type plants (Fig. 1B). To compare the transcript level of SSPs and non-SSPs, we measured the transcript levels in developing seeds. The levels of *12S1*, *12S3* and *12S4* transcripts relative to that of *UBQ10* were 3.76, 0.30 and 0.97, respectively, in developing wild-type (WT) seeds at 15 days after flowering (DAF) (Table 1). Although the transcripts were relatively abundant in developing seeds, their levels were comparable to those of *ISOCITRATE LYASE* (*ICL*) and *LATE EMBRYOGENESIS ABUNDANT 1* (*LEA1*), which encode proteins with lower levels of accumulation in seeds (Table 1). These results demonstrate that there is a low correlation between the levels of transcripts and proteins, suggesting that the predominant accumulation of SSPs may result from post-transcriptional regulation.

**Table 1:** The number of SSP transcripts in 15 DAF *Arabidopsis* seeds.

Genes	The number of transcripts/*UBQ10* transcript* (Mean ± SE)	The protein content^†^ (% of total seed protein)
SSPs		
*12S1*	03.76 ± 0.32	40
*12S3*	00.30 ± 0.02	8.5
*12S4*	00.97 ± 0.03	34
Non-SSPs		
*ICL*	00.87 ± 0.09	< 1.0
*LEA1*	03.63 ± 0.34	< 1.0

*The expression level of each gene was measured by qRT-PCR and the number of transcripts per *UBQ10* transcript was estimated from the standard curve generated by the plasmid containing the coding sequence of each gene. The primer sets for the quantitative real-time PCR are shown in Table S1.

^†^Total protein content reported by Higashi et al*.*^22^.

### 3’UTRs of SSP genes are essential for their predominant accumulation

**Figure 2:**
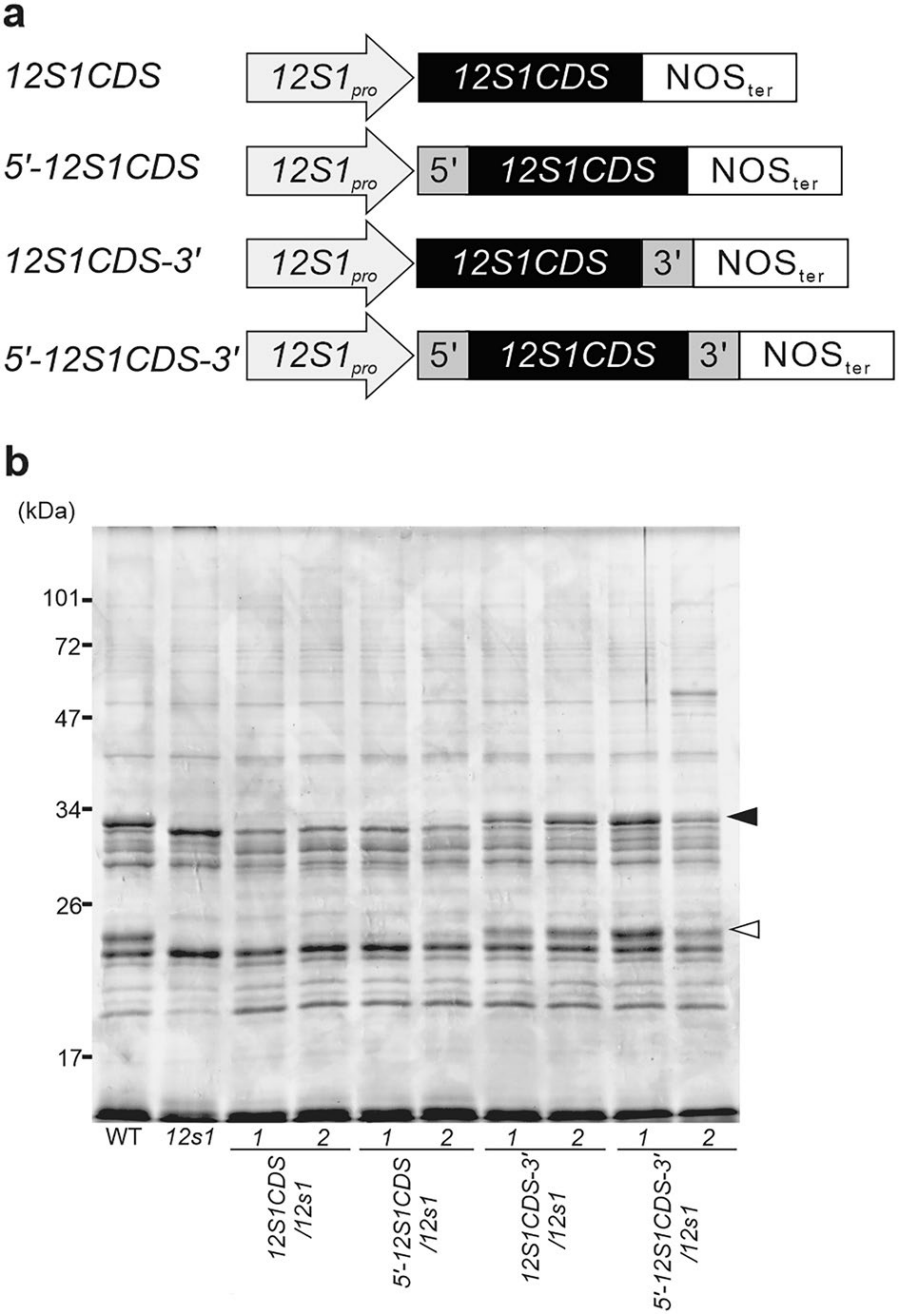
The 3’UTR is essential for 12S1 protein accumulation in *Arabidopsis* seeds. (**a**) Schematic representation of constructs of *12S1* with and without the 5’UTR and/or 3’UTR. *12S1CDS* indicates the coding sequence of *12S1*; 5’ indicates the 5’UTR of *12S1*; 3’ indicates the 3’UTR of *12S1*; NOSter indicates the nopaline synthase terminator. (**b**) CBB staining of seed proteins from two independent transgenic lines of *12S1CDS/12s1*, *5’-12S1CDS1/12s1*, *12S1CDS-3’/12s1*, and *5’-12S1CDS-3’/12s1*. Total protein was extracted using SDS sample buffer containing 10% (v/v) 2-mercaptoethanol. Total protein (10 µg) was loaded onto each well of a 12.5% polyacrylamide gel and protein bands were visualized by CBB staining. The 12S1 protein was separated into alpha (12S1α, closed arrowhead) and beta (12S1β, open arrowhead) subunits using a reducing agent in the buffer. *12s1* is the knockout mutant of the *12S1* gene.

**Figure 3:**
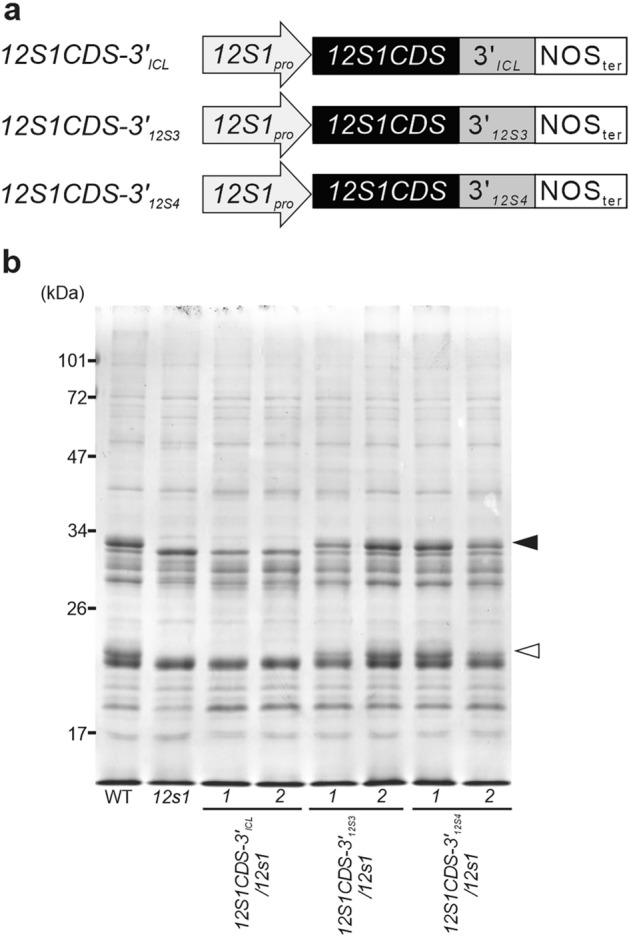
The role of the 3’UTRs of SSPs in protein accumulation in seeds. (**a**) Schematic representation of *12S1* constructs fused with the 3’UTR of *ICL*, *12S3* and *12S4*. (**b**) CBB staining of seed proteins from two independent transgenic lines of *12S1CDS-3’*_*ICL*_*/12s1*, *12S1CDS-3′*_*12S3*_*/12s1* and *12S1CDS-3′*_*12S4*_*/12s1*.

During seed development, the central vacuole rapidly disappears and protein storage vacuoles appear. Proteolytic activity is much lower in seeds than in other tissues, suggesting that low protein degradation is a reason for the accumulation of SSPs at a high level. Additionally, we hypothesized that the accumulation of SSPs is post-transcriptionally controlled because the transcripts of SSPs are comparable to those of low abundant proteins in seeds. We focused on the UTRs of SSP transcripts and investigated their role in protein accumulation. We prepared the coding sequence of *12S1* (*12S1CDS*), the coding sequence fused with the 5’UTR of *12S1* (*5’-12S1CDS*), the coding sequence fused with the 3’UTR of *12S1* (*12S1CDS*-3’), and the coding sequence fused with both the 5’UTR and 3’UTR of *12S1* (5’-*12S1CDS*-3’), and introduced these sequences (under the control of the *12S1* promoter) into *12s1-*knockout plants (Fig. 2A). Accumulation of the 12S1α and β subunits was fully recovered in transgenic plants harboring *12S1CDS-3’* and *5’-12S1CDS-3’*, but was not recovered in *12S1CDS* or *5’-12S1CDS* plants (Fig. 2B). Expression of *12S1* was recovered in all transgenic plants (Fig. S1). These results indicate that the 3’UTR of *12S1* is essential for the predominant accumulation of 12S1 protein in *Arabidopsis* seeds. Additionally, we investigated whether the 3’UTRs of other SSP genes (*12S3*, *12S4*) play a similar role. In addition, we introduced *12S1*CDS fused with the 3’UTR of an SSP gene or the 3’UTR of isocitrate lyase (*ICL*), a non-SSP gene, under the control of the *12S1* promoter into *12s1*-knockout plants (Fig. 3A). Introduction of *12S1CDS-3′*_*12S3*_ or *12S1CDS-3′*_*12S4*_ resulted in the recovery of 12S1 protein accumulation in *12s1*-knockout plants, while introduction of *12S1CDS-3’*_*ICL*_ did not (Fig. 3B). These results demonstrate that the 3’UTRs of SSP genes specifically function as a determinant for the predominant accumulation of SSPs.

### Fusion with the 3’UTRs of SSPs enables massive accumulation of recombinant pMDH1 protein

**Figure 4:**
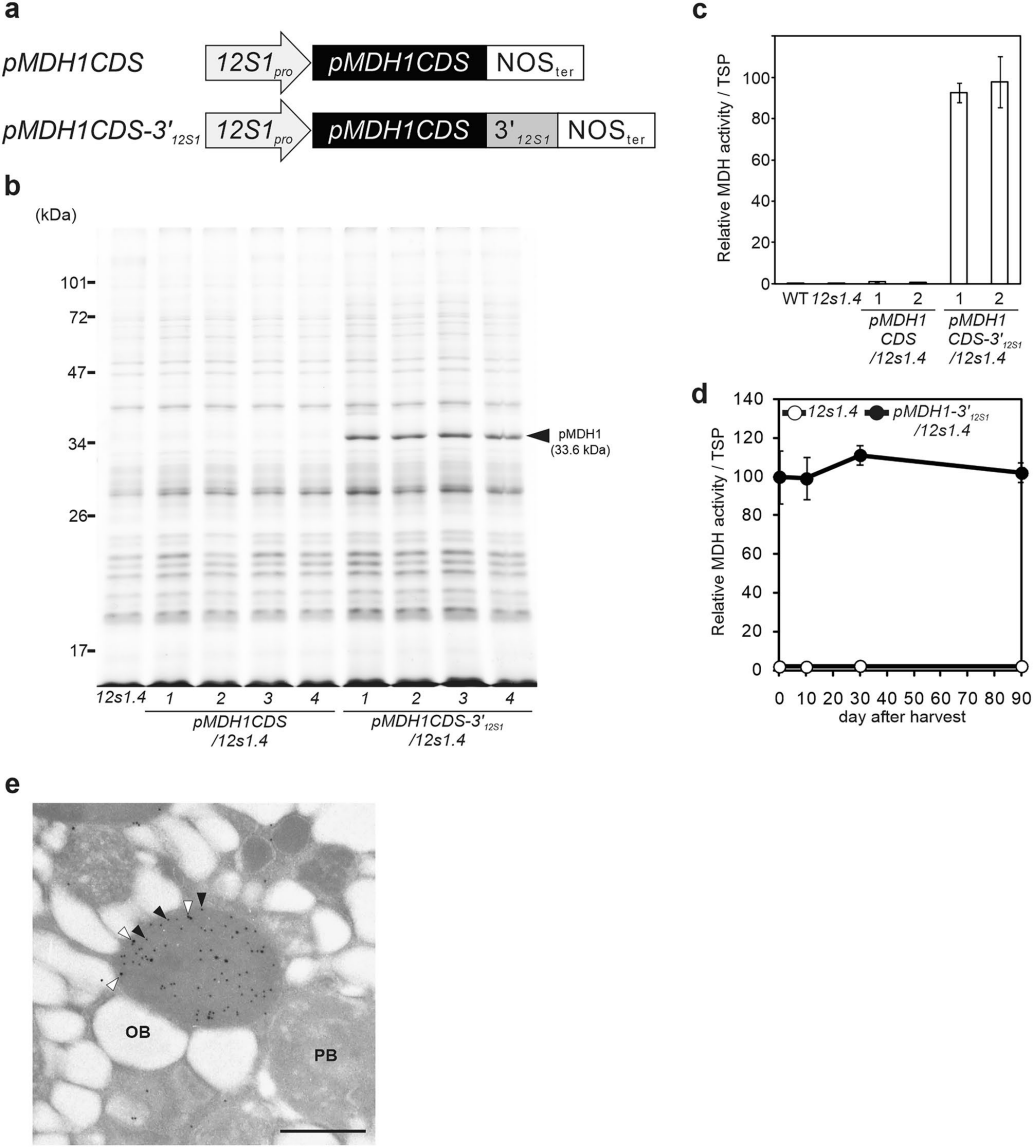
Massive accumulation of pMDH1 protein using the 3’UTR of *12S1*. (**a**) Schematic representation of *pMDH1CDS* fused with or without 3′_*12S1*_. *pMDH1CDS* represents the coding sequence of the *pMDH1* gene. (**b**) CBB staining of seed proteins from four independent transgenic lines of *pMDH1CDS/12s1.4* and *pMDH1CDS-3′*_*12S1*_*/12s1.4*. *12s1.4* is the knockout mutant of both *12S1* and *12S4*. The arrowhead indicates pMDH1 bands. (**c**) MDH activity in seeds of the transgenic lines. The activity of MDH/Total soluble protein (TSP) is expressed relative to the activity of WT. Values are presented as the mean ± SE of four independent experiments. (**d**) Enzymatic activity of pMDH1 in seeds stored at room temperature. Seeds of *12s1.4* and pMDH1-3′_*12S1*_/*12s1.4* were collected in 15 ml plastic tubes and stored at room temperature (22–28 °C) under dark conditions. Values are the mean ± SE of four independent experiments. (**e**) Immunoelectron micrograph of *pMDH1CDS-3′*_*12S1*_*/12s1.4* seeds. Localizations of catalase and pMDH1 are indicated by anti-catalase antibody-conjugated 25 nm gold particles (open arrowheads) and anti-pMDH1 antibody-conjugated 15 nm gold particles (closed arrowheads). OB, oil body; PB, protein body. Scale bar, 1.0 µm.

To verify whether the control of protein accumulation by the 3’UTRs of SSPs applies to other proteins, we fused the 3’UTR of *12S1* to non-SSPs. In addition, reducing endogenous SSP levels increases the accumulation of exogenous recombinant proteins in rice^12^ and *Arabidopsis*^11^, we generated a *12S1* and *12S4* (*12s1.4*) double knockout line (Fig. 1B) for use in subsequent experiments. We introduced *pMDH1*, encoding peroxisomal malate dehydrogenase 1 in *Arabidopsis thaliana*, with or without the 3’UTR of *12S1* at its 3’ end (*pMDH1CDS-3’UTR*_*12S1*_ and *pMDH1CDS*, respectively) under the control of the *12S1* promoter into *12S1.4* (Fig. 4A). Gene expression of *pMDH1* was confirmed in developing seeds of both *pMDH1CDS/12s1.4* and *pMDH1-3’UTR*_*12S1*_*/12s1.4* plants (Fig. S2). In the seeds of *pMDH1CDS-3’UTR*_*12S1*_*/12s1.4* plants, pMDH1, like SSPs, represented a major protein, whereas this protein was not detected in *pMDH1CDS/12s1.4* seeds (Fig. 4B). In the seeds of *pMDH1-3’UTR*_*12S1*_*/12s1.4* plants, pMDH1 represented approximately 40% of the total protein content. We measured the enzymatic activity of MDH in the seeds of WT, *12s1.4*, *pMDH1CDS/12s1.4*, and *pMDH1-3’UTR*_*12S1*_*/12s1.4* plants. The MDH activity of *pMDH1CDS/12s1.4* seeds was eightfold higher than that of WT seeds, while the activity of *pMDH1CDS-3’UTR*_*12S1*_/*12s1.4* seeds was approximately 100-fold higher than that of *pMDH1CDS/12s1.4* seeds (Fig. 4C). These results demonstrate that fusion of the 3’UTR of *12S1* greatly enhances the accumulation of recombinant proteins. Fig. S3 showed GFP accumulation in another example of recombinant protein accumulation. Significant GFP accumulation and high intensity of fluorescence were observed only when the 3' UTR of 12S1 is fused. These results indicate that the enhancement of recombinant protein accumulation by fusion of the *3' UTR* of *12S1* is independent of the origin of the gene encoding the recombinant protein or the protein tag sequence for purification and labeling. Moreover, the MDH activity remained consistent in seeds stored at room temperature for at least 90 days (Fig. 4D). These results demonstrate that fusion of the 3’UTR of *12S1* enables massive accumulation of pMDH1, a non-SSP, in dry seeds, and that this protein maintains its enzymatic activity. Moreover, it is reported that the subcellular localization of pMDH1 was peroxisomal^24^. Indeed, immunoelectron microscopic analysis also revealed that pMDH1 stained by anti-pMDH1 antibody-conjugated 15 nm gold particles co-localized with catalase, a marker protein for peroxisomes (which were stained by anti-catalase antibody-conjugated 25 nm gold particles; Fig. 4E). These results indicate that pMDH1 localized to the peroxisomes in seeds of *pMDH1CDS-3’UTR*_*12S1*_*/12s1.4*. The results also suggest that fusion of the 3’UTRs of SSPs is a useful technique for the production of massive amounts of recombinant protein with the correct intracellular localization and activity.

### Fusion of the 3’UTRs of SSPs enables massive production of human IFN lambda-3 in *Arabidopsis* seeds

**Figure 5:**
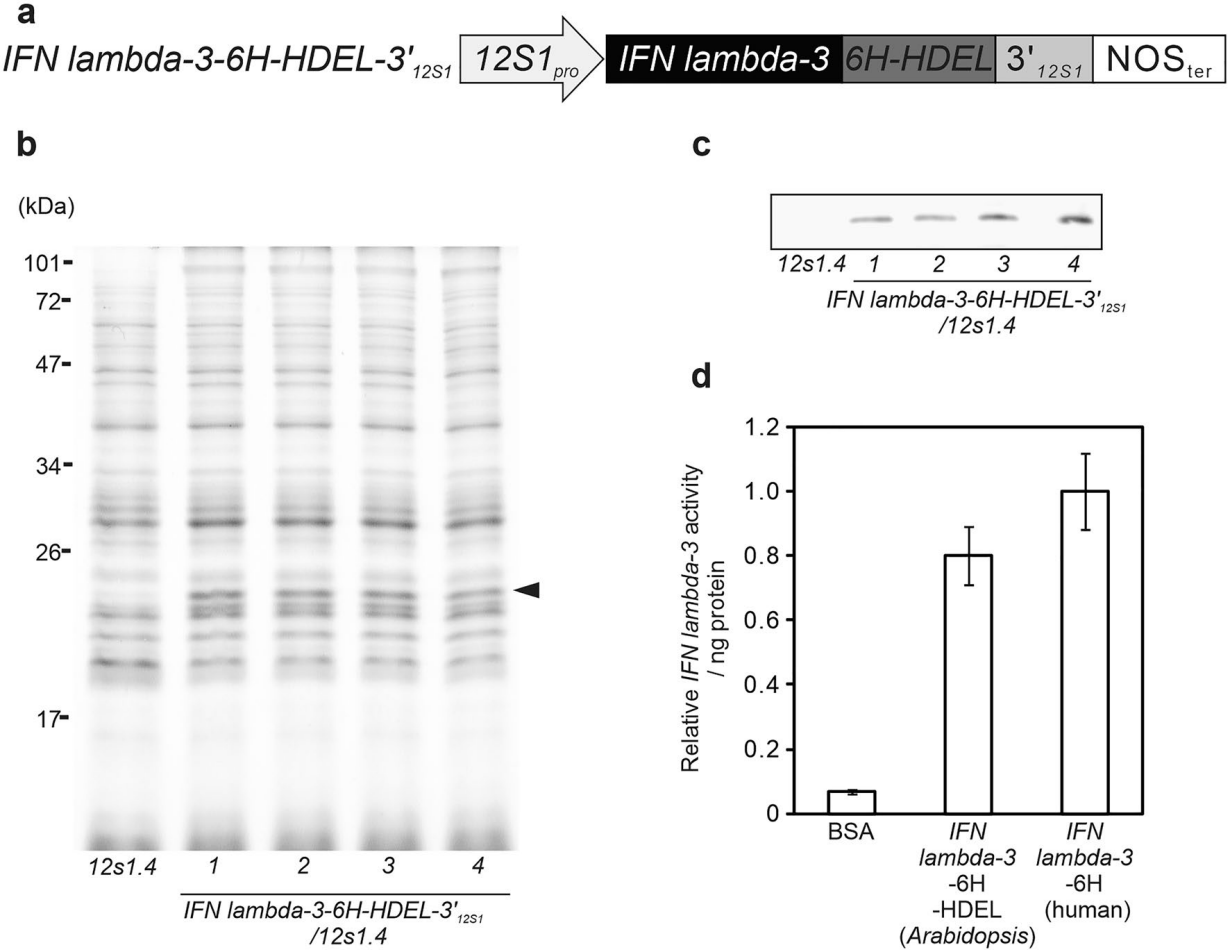
Production of IFN lambda-3 in *Arabidopsis* seeds. (**a**) Schematic representation of recombinant *IFN lambda-3* fused with 3′_*12S1*_. *IFN lambda-3CDS* represents the coding sequence of the *IFN lambda-3* gene. 6 × His-HDEL represents the ER-retention signal (HDEL) fused with a 6 × Histidine tag. (**b**) CBB staining of seed proteins from WT, *12s1.4*, and four independent transgenic lines of *IFN lambda-3–6* × *His-HDEL-3′*_*12S1*_*/12s1.4*. The arrowhead indicates IFN lambda-3 bands. (**c**) Immunoblot analysis of seed protein from WT, *12s1.4*, and *IFN lambda-3–6* × *His-HDEL-3′*_*12S1*_*/12s1.4* plants. (**d**) Relative activity of purified recombinant IFN lambda-3. IFN lambda-3 activities are relative to the activity of recombinant IFN lambda-3 produced in human cells. Values are presented as the mean ± SE of four independent experiments.

Biopharmaceuticals against cancer and infectious diseases, such as interferons, are needed worldwide. Plant-based protein production methods represent a promising approach to biopharmaceutical production due to the low risk of endotoxin or infectious agent contamination from serum and the low production costs. To confirm that our method is applicable to the production of interferons, we produced human IFN lambda-3, a candidate biopharmaceutical agent for treating hepatitis C infection^25,26^, in *Arabidopsis* seeds by fusing this gene with the 3’UTR of *12S1*. Based on previous reports^15,16,27^, an endoplasmic reticulum (ER)-retention signal (HDEL) was fused to the C-terminal end of *IFN lambda-3BCDS* (Fig. 5A). IFN lambda-3 accumulated to high levels in seeds of the transgenic plants (Fig. 5B,C), representing approximately 14% of the total protein content in seeds. The activity of IFN lambda-3 was measured in a luciferase assay using HepG2 cells derived from human liver. The HepG2 cells were cultured for 24 h in a medium containing 100 ng/ml of IFN lambda-3 produced with *Arabidopsis* seeds, IFN lambda-3 produced with human embryonic kidney cells or BSA. IFN lambda-3 purified from *Arabidopsis* seeds had activity levels similar to that of IFN lambda-3 produced in human cells (Fig. 5D).

## Discussion

SSPs represent the major proteins that accumulate in seeds. Therefore, the mechanisms underlying SSP accumulation have been studied to increase recombinant protein production. In seeds, SSPs are transported from ER to (and accumulate in) protein bodies; the signal peptides of SSPs are responsible for their targeting into ER and subsequent in protein bodies^12,23,28^. Several recombinant proteins fused to this signal peptide accumulated in protein bodies to higher levels than those not fused to the signal peptide^12,15,29–31^, demonstrating that the intracellular localization of proteins contributes to protein yields. However, the levels of recombinant proteins in these studies were considerably lower than those of SSPs in most case, especially in *Arabidopsis* seeds, which indicates that a key factor other than intracellular localization is required for the predominant accumulation of SSPs. Indeed, in our study, deletion of the 3’UTR of *12S1* significantly reduced the accumulation of 12S1 (Fig. 2B), and addition of the 3’UTR dramatically increased the levels of pMDH1 and human IFN lambda-3 and GFP in seeds (Figs. 4B, 5B and S3). These results clearly show that the 3’UTR of SSPs is the key factor determining the predominant accumulation of SSPs in *Arabidopsis* seeds. The pMDH1 protein fused to the 3’UTR correctly localized to peroxisomes (Fig. 4E), suggesting that the 3’UTR enhances the accumulation of target proteins without altering their intracellular localization. In developing seeds of transgenic plants, the transcripts of the transgenes were abundant with or without the 3’UTR (Figs. S1 and S2), suggesting that the 3’UTR is involved in increasing protein accumulation by post-transcriptional regulation. Recent studies have revealed that the 3’UTR is involved in mRNA stability and translation^32,33^. In rice, prolamine and glutelin mRNAs are specifically localized to the protein body ER and cisternal ER, respectively, and the 3’UTRs of these mRNAs are involved in mRNA localization^17–19^. Although the 3’UTRs of rice SSPs are important for mRNA localization, they have a relatively minor effect on SSP accumulation^21^. As the addition of the 3’UTR of *Arabidopsis* SSPs greatly enhanced protein accumulation, the function of the 3’UTRs of SSPs may be fundamentally different between rice and *Arabidopsis*. Intracellular localization may represent the key factor in the predominant accumulation of SSPs in rice seeds, whereas the presence of the 3’UTR is the key factor in *Arabidopsis* seeds. To date, the molecular functions of the 3’UTRs remain unclear compared with those of the 5’UTRs. Further studies in various organisms are needed to obtain a better understanding of this important genetic region.

Seed-based production systems for recombinant proteins have recently been reported^7,8,34^. In those systems, they employed ER and protein bodies to enhance recombinant protein yield; the recombinant proteins are fused with SSP signal peptides and ER-retention signals to their localization, yet the recombinant protein levels were lower than those of SSPs. By contrast, in the current study, the fusion of the 3’UTR of SSPs enabled massive accumulation of pMDH1, despite the absence of SSP signal peptides and ER-retention signals (Fig. 4A,B). These results indicate that fusion of the 3’UTR is a promising, novel method to produce recombinant proteins. This method, which does not alter the intracellular localization of recombinant proteins, leads to the production of foreign proteins with the correct intracellular localization under near-native cellular conditions. In fact, pMDH1 produced in the *Arabidopsis* seeds was correctly localized to the peroxisome and had MDH activity (Fig. 4C,E). These results indicate that this method is useful for producing recombinant proteins for life science research, providing high yields and activity. In addition, this method is promising to produce biopharmaceuticals due to the low risk of endotoxin or infectious agent contamination from serum and the low production costs. High levels of IFN lambda-3, a biopharmaceutical candidate for treating hepatitis C infection, were produced in seeds, with adequate activity (Fig. 5B,E). Previously, the yield of human interleukin-10 produced in *Arabidopsis* seeds was approximately 0.02% of total soluble protein levels in seeds^16^ and that of murine interleukin-10 was 0.7%^15^. The content of IFN lambda-3 produced by our method was 14% of total soluble protein in seeds, indicating that fusion of the 3’UTR of *12S1* greatly increases interferon production in *Arabidopsis* seeds. Yang et al. (2012) increased the yield of IL-10 in rice to 220 ng/grain, corresponding to approximately 1% of seed weight^12^. Since the protein content of *Arabidopsis* seeds is approximately 30%, the yield of IFN lambda-3 produced by our method was approximately 4.2% of seed weight. Compared with rice, *Arabidopsis* is easy to grow indoors, requiring less soil and fewer fluorescent lamps. In addition, it takes only 2 months to germinate plants and harvest new seeds from *Arabidopsis*, and the transformation process of *Arabidopsis* (Agrobacterium floral dip method) is simpler than that of other plants (transformation via callus and regeneration of plants)^35,36^. Considering the low cost, ease, and rapid growth of *Arabidopsis*, our method is applicable to interferon production, and it represents one of the most effective methods for biopharmaceutical manufacturing using plant species.

## Material and methods

### Plant materials

*Arabidopsis thaliana* Col-0 strain was used as the WT. All seeds were surface-sterilized in 2% (w/v) NaClO (sodium hypochlorite) and 0.02% (v/v) Triton X-100. Seeds were sown on growth medium containing half-strength Murashige and Skoog salts, Gamborg’s B5 vitamins, 0.5 mg/ml MES, 0.8% (w/v) agar, and 1% (w/v) sucrose. The pH of the medium was adjusted to 5.8 with KOH. Two-week-old plants were transferred to soil under long-day conditions (16 h light/8 h dark) at 22 °C. The *Arabidopsis* T-DNA insertion mutant collections are useful resources for genetic studies and are used by many researchers. The T-DNA insertion line of *12s1* was obtained from the European *Arabidopsis* Stock Centre at the University of Nottingham, Sutton Bonington Campus, UK (GK-283D09.01). The Col-0 and the T-DNA insertion lines of *12s2*, *12s3*, and *12s4* were obtained from the *Arabidopsis* Biological Resource Center based at Ohio State University, USA (SALK_049575, SALK_085866, and SALK_002668, respectively). The *12s1.3* and *12s1.4* double knockout lines were established by crossing *12s1* with *12s3*, and *12s1* with *12s4*, respectively. Growth of the *12s1.4* was comparable to the WT, at least, under the laboratory conditions. It has been reported that a number of mutants with T-DNA insertions in intron sequences show phenotypes similar to those of knockouts^37^. *12s1.3.4* triple knockout line could not be established. The names of the *12S1*, *12S2*, *12S3* and *12S4* genes encoding 12S seed storage proteins by UniProt are *CRC*, *CRD*, *CRB* and *CRA1*, respectively. Experimental studies on plants, including the collection of plant material, were conducted in accordance with relevant institutional, national and international guidelines and legislations.

### Plasmid construction and plant transformation

A series of *12S1* fragments, coding sequences of *pMDH1* (NCBI Reference Sequence: NM_127843.5), and were amplified from *Arabidopsis* cDNA by PCR. The coding sequence of *IFN lambda-3* (GenBank: AY129149.1) was amplified from cDNA from Raji, a human T cell leukemia cell line, by PCR. The 3’UTR of SSPs and *6* × *His-HDEL* were fused by fusion PCR. These DNA fragments were cloned into pDONR221 with Gateway® cloning technology (Thermo Fisher Scientific, Massachusetts, USA). The promoter sequence 1671 bp upstream of the *12S1* gene was amplified from the *Arabidopsis* genome and cloned into pDONRP4P1R. The primer sets for the plasmid construction are shown in Table S1. The expression vectors were constructed to combine the *12S1* promoter from pDONR P4-P1R and DNA fragments from pDONR221 into R4pGWB501^38^ with Multisite Gateway® technology (Thermo Fisher Scientific, Massachusetts, USA). These expression vectors were transformed into *Agrobacterium tumefaciens* strain C58C1^rif^ and introduced into WT, *12s1*, or *12s1.4* lines using the floral dip method. Transformants were selected on medium containing 25 μg mL^−1^ hygromycin B. The accumulation of recombinant protein was evaluated in at least ten independent T3 progeny.

### SDS-PAGE and immunoblot analysis

To extract total proteins, 100 seeds of each line were ground using a stainless-steel pestle in 100 µl of SDS buffer containing 125 mM Tris–HCl (pH 7.5), 10% (v/v) 2-mercaptoethanol, 4% (w/v) SDS, and 10% (w/v) sucrose. The samples were boiled for 5 min and centrifuged at 10,000 g for 5 min. The supernatants were collected and the concentrations of total protein were measured by Bradford ULTRA™. The protein contents of the supernatants were adjusted to 0.5 μg/μl by adding SDS buffer and used as protein samples. Protein samples extracted from seeds were separated in 12.5% or 15.0% SDS–polyacrylamide gels. Protein bands were detected with CBB-R250 staining or transferred to Immobilon-P PVDF membranes (Merck Millipore, Massachusetts, USA) using semidry electroblotting for immunoblot analysis. Monoclonal anti- IFN lambda-3 antibody (MAB5259, R&D Systems, Minneapolis, USA) was used in the immunoblot analysis. The PVDF membrane was cut out to include the region of interest and immuno-reactive protein bands were detected using the SNAP i.d. (Merck Millipore, Massachusetts, USA) and ECL systems (

Cytiva, Tokyo, Japan). The recombinant protein levels were estimated based on the intensities of the protein bands in SDS–polyacrylamide gels using MultiGauge Ver3.0 (Fujifilm, Tokyo, Japan). The uncropped blots are shown in Fig. S4.

### Electron microscopy

Ultrathin sections were prepared from dry seeds of WT and transgenic lines. Sectioning, immunogold labeling, and microscopic observation were performed as described previously^39^.

### MDH activity

One hundred seeds of WT or transgenic lines were ground using a stainless-steel pestle in 200 µl of buffer containing 100 mM KH_2_PO_4_ (pH 8.0) on ice. The samples were centrifuged at 10,000 g for 5 min at 4 °C. After removing the oil layer, the supernatants were carefully collected and used as crude extracts. The contents of total soluble proteins (TSP) of the crude extracts were measured by Bradford ULTRA™. MDH activities were measured according to the previous protocol^40^. A standard curve was generated using MDH from porcine heart (M-7383, Sigma-Aldrich, Missouri, USA).

### Purification of recombinant IFN lambda-3 from seeds

One hundred seeds of *IFN lambda-3–3′*_*12S1*_ were ground using a stainless-steel pestle in 500 µl of lysis buffer containing 50 mM NaH_2_PO_4_ (pH 8.0), 300 mM NaCl, and 10 mM imidazole on ice. The samples were centrifuged at 10,000 g for 10 min at 4 °C. After removing the oil layer, the supernatants were carefully collected and loaded onto a Ni–NTA spin column (QIAGEN, Venlo, the Netherlands) and centrifuged at 300 g for 10 min at 4 °C, and the flow-through was discarded. The spin column was washed four times with 600 µl of wash buffer containing 50 mM NaH_2_PO_4_ (pH 8.0), 300 mM NaCl, and 20 mM imidazole, followed by centrifugation at 800 g for 2 min at 4 °C. The recombinant IFN lambda-3 protein was eluted with 100 µl of elution buffer containing 50 mM NaH_2_PO_4_ (pH 8.0), 300 mM NaCl, and 500 mM imidazole, followed by centrifugation at 800 g for 5 min at 4 °C (shown in Fig. S5).

### Evaluation of IFN lambda-3 activity using luciferase assays

Luciferase assays of recombinant protein were performed using a Dual-Glo Luciferase reporter assay system (Promega, Wisconsin, USA). To assess recombinant protein activity, HepG2 cells were transfected with pISRE-Luc and pGL4.74, and harvested 24 h after treatment with purified recombinant interferon IL28B from seeds. Chemiluminescence was measured by SpectraMax L (Molecular Devices, California, USA). Firefly luciferase activity was normalized to *Renilla* activity to adjust for transfection efficiency. Bovine serum albumin (100 ng/mL) and recombinant human IFN lambda-3 produced by 293F cells, derived from human embryonic kidney cells^25^, were used as the negative and positive controls, respectively.

## Supplementary Information

Supplementary Information.

## Data Availability

All data and materials appearing in this study are available from the corresponding author (M.K.) upon reasonable request.
