# Supplementary material for: Fusing the 3’UTR of seed storage protein genes leads to massive recombinant protein accumulation in seeds
Source: Sci Rep. 2023 Jul 27;13:12217. doi: 10.1038/s41598-023-39356-3 (PMC10374616; doi:10.1038/s41598-023-39356-3)
Supplement: Supplementary file 1 — Supplementary Information. [file 41598_2023_39356_MOESM1_ESM.pdf]

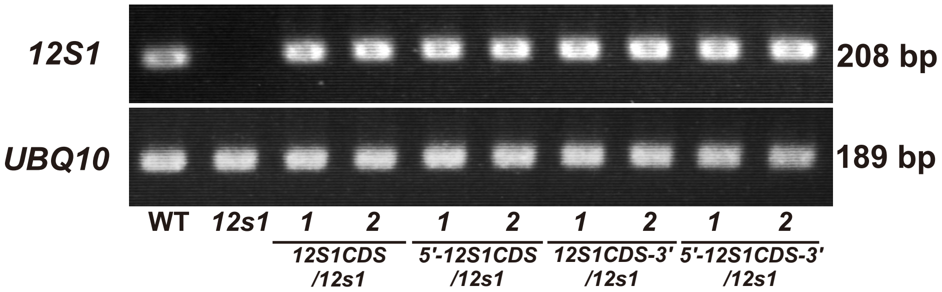

Fig. S1. Confirmation of gene expression of *12S1* in the transgenic plants.

Total RNA was extracted from 15 DAF seeds of WT, *12s1*, and two independent lines of the transgenic plants. The cDNA was synthesized from 1 μg of the total RNA and the concentration of the cDNA was adjusted at 2 ng/μl. After PCR, amplicons of *12S1* and *UBQ10* were separated in a 2.5 % agarose gel. The primer sets used in this experiment are listed in Table S1.

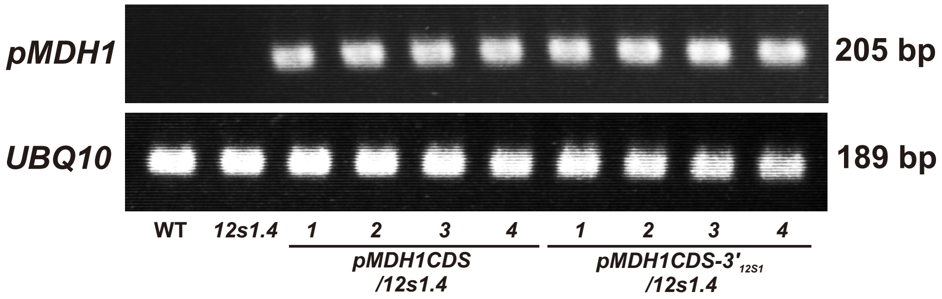

Fig. S2. Confirmation of gene expression of *pMDH1* in the transgenic plants.

Total RNA was extracted from 15 DAF seeds of WT, *12s1*, and four independent lines of the transgenic plants. cDNA synthesized from 1 μg of the total RNA and the concentration of the cDNA was adjusted at 2 ng/μl. After PCR, amplicons of *pMDH1* and *UBQ10* were separated with 2.5 % agarose gel. The primer sets used in this experiment are listed in Table S1.

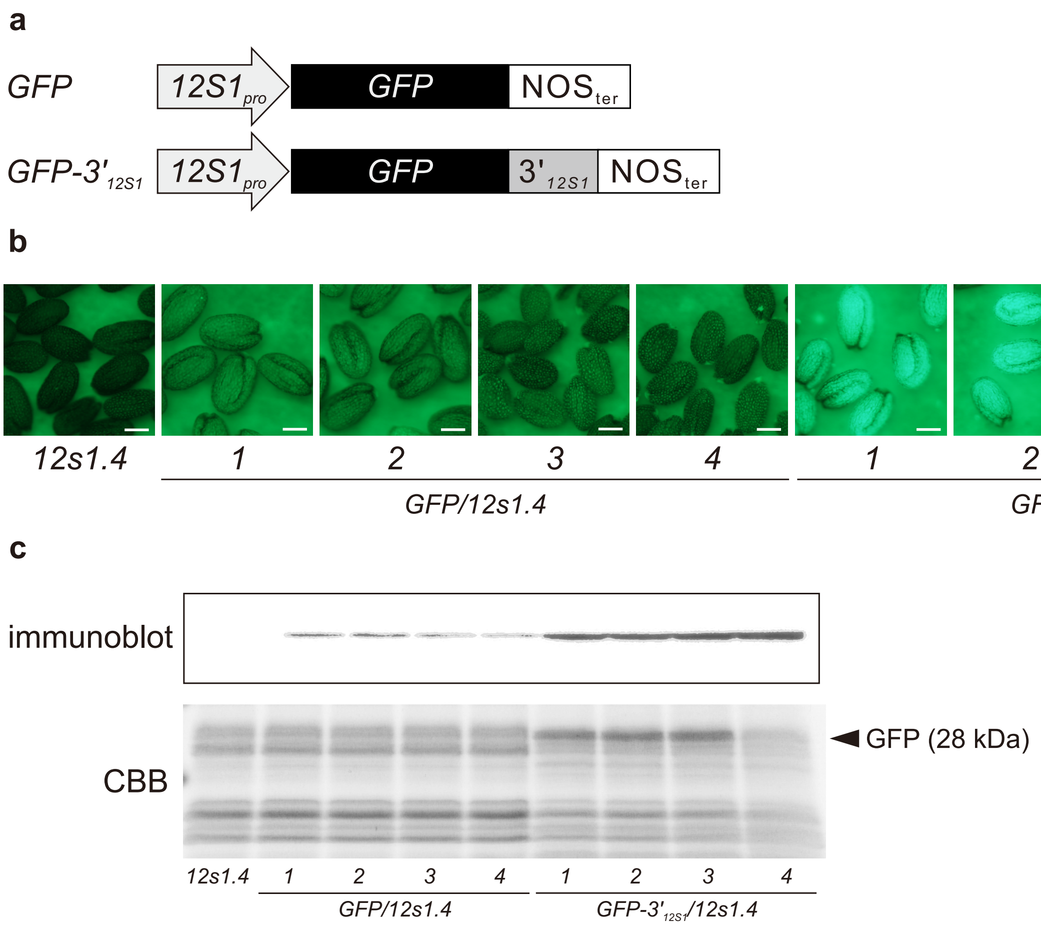

Fig. S3. Accumulation of GFP using the 3'UTR of *12S1*.

(a) Schematic representation of *GFP* fused with or without 3'$_{12S1}$. *GFP* represents the coding sequence of the *green fluorescence protein* gene from R4pGWB504. (b) Detection of GFP fluorescence in dry seeds. Scale bars represent 200 µm. (c) Immunoblot and CBB staining of seed proteins from four independent transgenic lines of *GFP/12s1.4* and *GFP-3'*$_{12S1}$*/12s1.4*. *12s1.4* is the knockout mutant of both *12S1* and *12S4*.The arrowhead indicates GFP bands. Monoclonal anti-GFP (1E4, MBL, Nagoya, Japan) was used in the immunoblot analysis.

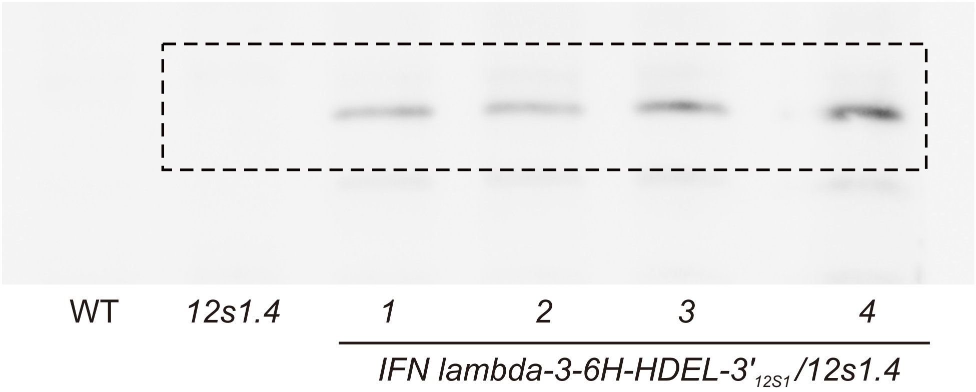

Fig. S4. Uncropped western blot images for Figure 5c.

Dashed boxes indicate areas that were cropped.

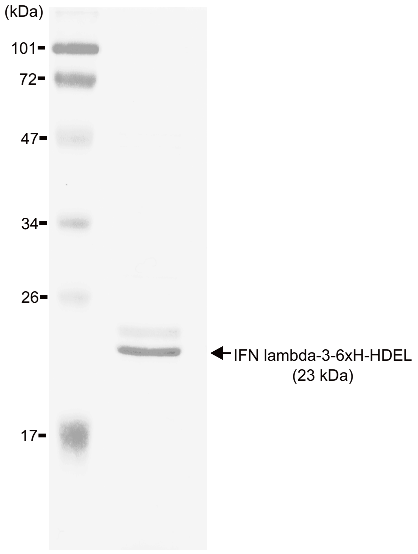

Fig. S5. Purified IFN lambda-3 in *Arabidopsis* seed by Ni-NTA column.

Recombinant IFN lambda-3 protein (2.5 μg) was loaded onto each well of 12.5 % SDS-polyacrylamide gel. Protein bands were visualized with CBB staining.

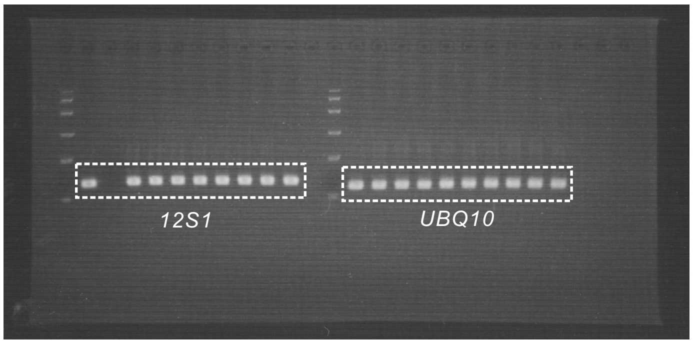

Fig. S6. Uncropped gel images for Figure S1.
Dashed boxes indicate areas that were cropped.

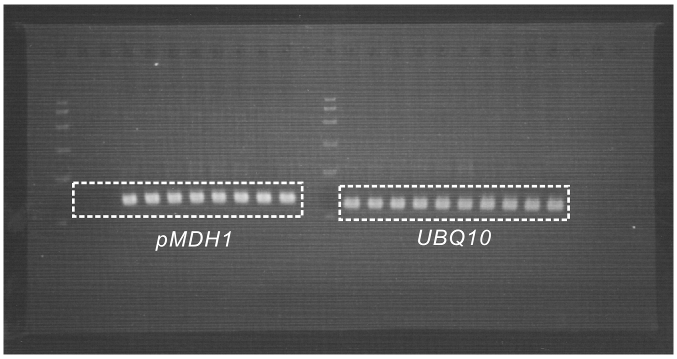

Fig. S7. Uncropped gel images for Figure S2.

Dashed boxes indicate areas that were cropped.

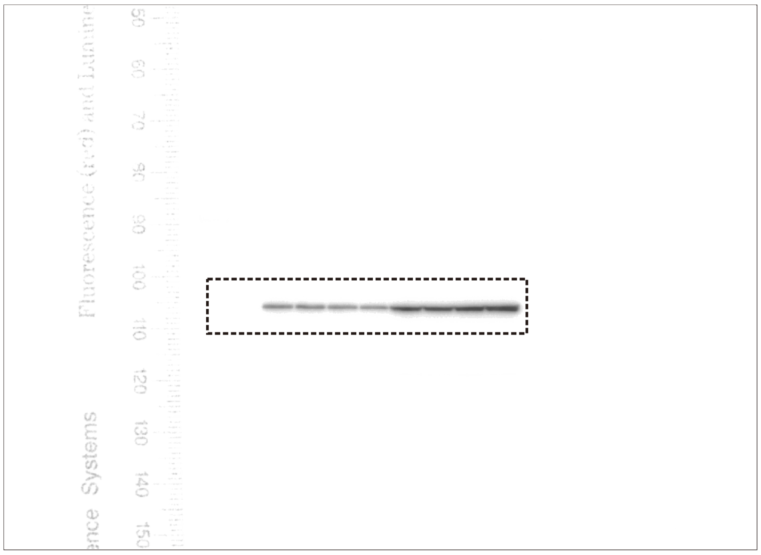

Fig. S8. Uncropped western blot images for Figure S3c.

Dashed boxes indicate areas that were cropped.

Table S1. Primer sequences used in this study.

Primer name	5'-sequence-3'
B4-12S1pro_F	ATAGAAAAGTTGTTGTCGAGCACGAACGTCATAG
B1R-12S1pro_R	TTTGTACAAACTTGCGACTTAGGAGGAGTTTATATATTG
B1-12S1CDS_F	AAAAAGCAGGCTTCATGGTTAAGCTCAGCAATCTCC
B1-12S1CDS_R	AGAAAGCTGGGTCTTAAGCCTCGACAATCTCCTC
B1-5’-12S1CDS_F	AAAAAGCAGGCTTCCCGTTCTCTTCATCCATCTCTC
B2-12S1CDS-3’_R	AGAAAGCTGGGTCAGACGAAAAAACTGGCGCTATA
12S1CDS-3’(ICL)_F	GAGGAGATTGTCGAGGCTTAAGACATATGTTCATTACGGCGGTG
B2-3’(ICL)_R	AGAAAGCTGGGTCAGAAACTGAATCACGAATATTATAAG
12S1CDS-3’(12S3)_F	GAGGAGATTGTCGAGGCTTAAGGCTTGATGAGCGCGTGG
B2-3’(12S3)_R	AGAAAGCTGGGTCAGAAATTAAAGACTTAAAGATTTTTTATTC
12S1CDS-3’(12S4)_F	GAGGAGATTGTCGAGGCTTAAGAGCTTAAAACTGCAGCTTAAC
B2-3’(12S4)_R	AGAAAGCTGGGTCAAGAACCTAATTTAGACCTAGATTTG
B1-pMDH1CDS_F	AAAAAGCAGGCTTCATGGATCCAAACCAACGTATC
B2-pMDH1CDS_R	AGAAAGCTGGGTCTTATTTCTTCGCAAAGGTAACAC
pMDH1CDS_R	TTATTTCTTCGCAAAGGTAACAC
pMDH1CDS-3’(12S1)_F	CCTTTGCGAAGAAATAAATCAAAACGTTTTTCTTTTTCTTAATAAAG
B1-IFN lambda-3_F	AAAAAGCAGGCTTCATGAAACTAGACATGACCGGGGAC
IFN lambda-3-6H-HDEL_R	TTAAAGATCTTCATGGTGGTGATGGTGATGATGGACACACAGGTCCCCGCTG
6H-HDEL-3’(12S1)_F	CATCATCACCATCACCACCATGAAGATCTTTAAATCAAAACGTTTTTCTTTTTCTTAATAAAG
12S1Q_F	CCATGGCAAGGACGACAG
12S1Q_R	TCACATGTTCCACCTTCTGG
12S3Q_F	CCTCTTCAATGGCTACACTGC
12S3Q_R	CCGTGAACAACAAACGTGAG
12S4Q_F	TGGCTCGAGTCTCTTCTCTTC
12S4Q_R	GATGATGTAACGTGCAAAGGAG
ICLQ_F	GGTCGCAGCTACTCTGATCC
ICLQ_R	GAGCCAAGCCACTGATCTTC
pMDH1Q_F	ACCAACGTATCGCGAGAATC
pMDH1Q_R	TGAAGAACCGAAACCAAAGG
LEA1Q_F	TTGCTGAAGGAAGGAGCAAG
LEA1Q_R	CGCTCTCCACCAGATTTTTC
UBQ10Q_F	GAAGTGGAAAGCTCCGACAC
UBQ10Q_R	TTAGAAACCACCACGAAGACG
